# Transfer RNA expression, modification, and derived small RNAs in cancer biology and clinical potential

**DOI:** 10.3389/fimmu.2026.1733897

**Published:** 2026-07-03

**Authors:** Ming Luo, Ji’an Liu, Yang Su, Rao Fu, Xufeng Huang, Qi Wang, Jing Li, Zhengrui Li, Li Liu

**Affiliations:** 1 Department of General Surgery, First Affiliated Hospital of Huzhou Normal University, Huzhou, Zhejiang, China; 2 Shanghai Jiao Tong University School of Medicine, Shanghai, China; 3 Department of Oral and Maxillofacial Surgery, Shanghai Xuhui District Dental Center, Shanghai, China; 4 Department of Data Visualization, Faculty of Informatics, University of Debrecen, Debrecen, Hungary; 5 Faculty of Dentistry, University of Debrecen, Debrecen, Hungary; 6 Shanghai Stomatological Hospital & School of Stomatology, Fudan University, Shanghai, China

**Keywords:** aminoacyl-tRNA synthetase, therapeutic targets, transfer RNA, tRNA modifications, tRNA-derived fragments, tumor biomarkers

## Abstract

Transfer RNA (tRNA) is essential for protein synthesis and undergoes diverse chemical modifications that regulate its stability and function. Dysregulation of tRNA expression and modifications, along with the activity of tRNA-derived small RNAs (tsRNAs) and aminoacyl-tRNA synthetases, has been increasingly linked to cancer development, influencing tumor proliferation, metastasis, stress responses, and therapy resistance. Here we systematically review the biological features of tRNA and its modifications, elucidate their mechanistic roles in various cancers, and explore the emerging functions of tsRNAs and tRNA-modifying enzymes. We also assess the diagnostic and therapeutic potential of targeting tRNA pathways in oncology. These insights provide a foundation for advancing precision medicine approaches by exploiting tRNA-related mechanisms to improve cancer diagnosis and treatment outcomes.

## 1 Introduction

Transfer RNA (tRNA) plays a crucial role in the translation of genetic information into proteins (1), serving as the essential adaptors that facilitate the decoding of messenger RNA (mRNA) codons. The structural and functional integrity of tRNAs is significantly influenced by a myriad of post-transcriptional chemical modifications (2–4). These modifications, which can occur at various positions on the tRNA molecule, enhance its stability, functionality, and interaction with ribosomes and aminoacyl-tRNA synthetases (aaRS) (5). The modifications not only ensure the fidelity of protein synthesis but also adapt tRNA to respond to cellular conditions, thereby regulating gene expression and cellular metabolism (6). The complexity of tRNA modifications has been shown to be pivotal in maintaining homeostasis within cells, and disturbances in these processes are increasingly recognized as contributing factors to various diseases, including cancer (7).

In the context of cancer, the expression and modification patterns of tRNAs are notably altered. Cancer cells often exhibit dysregulated tRNA levels, which can lead to aberrant protein synthesis and promote tumorigenesis (8). For instance, the modifications at specific positions, such as the wobble position of tRNA or the anticodon loop, can directly affect the translation efficiency of oncogenes and tumor suppressor genes, thereby influencing cancer progression (9–16). Furthermore, the altered expression of tRNA-modifying enzymes has been implicated in the pathophysiology of various malignancies, suggesting that these enzymes could serve as potential therapeutic targets (6). The dynamic landscape of tRNA modifications, therefore, provides insights into the regulatory mechanisms underlying cancer biology and highlights the potential for tRNA-based strategies in cancer diagnosis and treatment.

Recent studies have also identified tRNA-derived small RNAs (tsRNAs), which are products of tRNA cleavage, as important regulators in cancer biology. These small RNAs, which include tRNA-derived stress-induced RNAs (tiRNAs) and tRNA fragments (tRFs), have been found to modulate various cellular processes, such as apoptosis, proliferation, and metastasis (17). The dysregulation of tsRNAs has been associated with several types of cancers, indicating their potential as biomarkers for cancer diagnosis and prognosis (18). For example, specific tsRNAs have been linked to cancer hallmarks, including resistance to therapy and the promotion of metastasis, underscoring their relevance in the clinical setting (19).

Moreover, understanding the mechanisms by which tRNA and its modifications influence tumor biology can lead to the development of novel therapeutic strategies. The investigation of tRNA-modifying enzymes and their regulatory pathways may uncover critical insights into the molecular underpinnings of cancer, paving the way for targeted interventions (20). For instance, inhibiting specific tRNA modifications or restoring normal tRNA expression levels could enhance the efficacy of existing cancer therapies or provide new avenues for treatment (21). As research continues to elucidate the multifaceted roles of tRNA and its modifications in cancer, it becomes increasingly clear that these molecules are not merely structural components of the translational machinery but are integral players in the complex network of cellular regulation and tumorigenesis.

In summary, tRNA and its modifications are emerging as key players in cancer biology, influencing protein synthesis and cellular fate. The aberrant expression and modification of tRNAs in cancer cells highlight their potential as biomarkers and therapeutic targets, warranting further exploration into their roles in tumorigenesis and progression. This review aims to systematically summarize the current understanding of tRNA functions in cancer, exploring their clinical applications and the implications for future research in this field. By integrating insights from recent studies, we can better appreciate the intricate relationship between tRNA biology and cancer, ultimately contributing to the development of innovative diagnostic and therapeutic strategies.

## 2 Fundamentals of tRNA biology

### 2.1 Structure and translational role of tRNA

tRNA is a pivotal molecule in the translation process, serving as the essential adaptor that decodes the mRNA sequence into corresponding amino acids during protein synthesis. The structure of tRNA is characterized by a well-conserved tertiary conformation, typically described as an L-shape, which arises from the folding of its cloverleaf secondary structure. This unique three-dimensional shape is crucial for its function, allowing tRNA to interact effectively with ribosomes and aminoacyl-tRNA synthetases. The anticodon loop of tRNA contains a specific three-nucleotide sequence that pairs with the complementary codon on mRNA, ensuring the correct incorporation of amino acids into the growing polypeptide chain. The structural integrity of tRNA is maintained through various intramolecular interactions, particularly between the D-arm and T-arm, which stabilize the overall conformation and facilitate the binding of amino acids (22, 23).

Moreover, tRNA molecules undergo extensive post-transcriptional modifications that significantly influence their stability, functionality, and interaction with other biomolecules. These modifications include methylation, pseudouridylation, and the addition of various chemical groups, which can enhance the structural stability of tRNA and modulate its interactions with ribosomes and aminoacyl-tRNA synthetases. For instance, the presence of modified nucleotides at specific positions can affect the flexibility of the tRNA molecule, thereby influencing its ability to accurately decode mRNA and participate in translation (24, 25). The diversity of tRNA modifications is not only essential for the proper functioning of tRNA but also plays a significant role in cellular stress responses and the regulation of gene expression, underscoring the multifaceted roles of tRNA beyond mere adapters in protein synthesis (7).

The interplay between the structural features of tRNA and its chemical modifications highlights the complexity of tRNA biology. Recent studies have shown that specific modifications can enhance the decoding efficiency of tRNAs, particularly under stress conditions, by facilitating the recruitment of translation factors and ribosomal subunits (26, 27). Additionally, the dynamic nature of tRNA structures allows them to adapt to various cellular environments, which is crucial for maintaining protein synthesis under fluctuating physiological conditions. As research continues to unveil the intricate mechanisms governing tRNA function, it becomes increasingly clear that understanding the structural and functional basis of tRNA is essential for elucidating its roles in health and disease, particularly in the context of cancer and other pathologies where tRNA dysregulation is implicated (28, 29).

In humans, tRNAs originate from two distinct genetic systems. Most tRNAs are encoded by nuclear genes and function in cytoplasmic protein synthesis, whereas a smaller subset is encoded by mitochondrial DNA and participates in mitochondrial translation (30–32). Although both groups share fundamental structural features, they differ in their biogenesis, modification patterns, and regulatory mechanisms (33). Unless otherwise specified, this review primarily focuses on nuclear-encoded cytoplasmic tRNAs and their associated modifications, regulatory enzymes, and derived small RNAs, which represent the major tRNA-related pathways currently implicated in cancer biology (34–36).

In conclusion, the structural and functional characteristics of tRNA are foundational to its role in translation and cellular regulation. The ongoing exploration of tRNA’s diverse modifications and their implications for tRNA function will undoubtedly enhance our understanding of molecular biology and may pave the way for novel therapeutic strategies targeting tRNA-related pathways in various diseases.

### 2.2 Major tRNA modifications and functional significance

**Figure 1:**
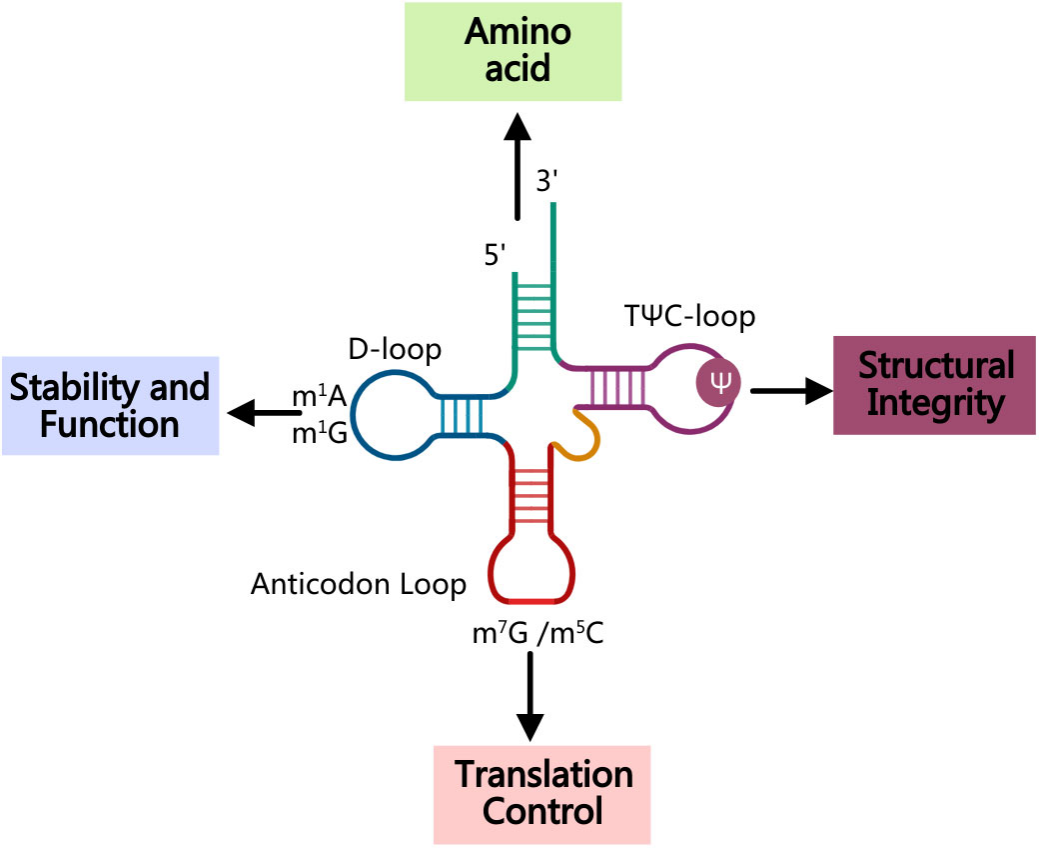
Major tRNA modifications and their functional significance.

tRNA modifications are crucial for the proper functioning of these molecules, which play a pivotal role in protein synthesis (37). Among the most common types of modifications are methylation, pseudouridylation, and the addition of N7-methylguanosine (m7G). Methylation occurs at various positions on the tRNA molecule, with modifications such as 2’-O-methylation and N6-methylation being particularly significant. For instance, the 2’-O-methylation of ribose sugars enhances tRNA stability and protects against degradation, while m7G modification at position 46 within the variable region of many tRNAs contributes to proper tRNA folding, stability, and translational efficiency has been shown to translational efficiency the initiation of translation and promote the stability of tRNA in the cellular environment (38). Pseudouridine (Ψ), another prevalent modification, is known to enhance the structural stability of tRNA by promoting proper folding and maintaining the integrity of the anticodon loop, thus facilitating accurate codon recognition during translation (39). The presence of these modifications not only stabilizes tRNA but also enhances its functionality by ensuring efficient and accurate decoding of mRNA codons, which is essential for protein synthesis (**Figure 1**). Importantly, tRNA modifications are not uniformly distributed across all tRNA species. Some modifications exhibit strong tRNA specificity and are associated with particular amino acid–decoding tRNAs (40, 41). For example, queuosine is primarily found in tRNAs recognizing Asp, Asn, His, and Tyr codons, whereas wybutosine is largely restricted to tRNAPhe. In contrast, modifications such as m7G and m5C occur across broader subsets of cytoplasmic tRNAs and contribute to global regulation of tRNA stability and translational efficiency (42).

Representative tRNA modifications, including m7G, m5C, Ψ, and wobble-base modifications, regulate tRNA stability, decoding accuracy, translational efficiency, and cellular stress adaptation.

The regulatory roles of tRNA modifications extend beyond mere structural integrity; they significantly impact the tRNA’s ability to interact with ribosomes and mRNA. For example, modifications such as queuosine (Q) at the wobble position of certain tRNAs are essential for maintaining translational fidelity and efficiency. Q-modification has been shown to enhance the decoding ability of tRNAs, particularly those that recognize codons for amino acids like tyrosine and asparagine (43). This modification allows for more precise pairing with mRNA codons, thereby minimizing errors during translation. Moreover, the presence of specific modifications can influence the kinetics of tRNA interactions with ribosomes, affecting the overall rate of protein synthesis (21). The dynamic nature of these modifications allows cells to adapt to varying metabolic conditions and stressors, highlighting their importance in maintaining cellular homeostasis.

Furthermore, the dysregulation of tRNA modifications has been implicated in various diseases, including cancer. Abnormalities in the expression of tRNA-modifying enzymes can lead to altered modification patterns, which in turn affect the stability and functionality of tRNAs. For instance, in several cancer types, the overexpression of tRNA methyltransferases has been associated with increased levels of m7G-modified tRNAs, which correlate with enhanced translation of oncogenic proteins (44). This suggests that cancer cells may exploit tRNA modification pathways to promote their growth and survival, making these modifications potential therapeutic targets. Understanding the precise mechanisms by which tRNA modifications influence translation and contribute to disease pathology could pave the way for novel treatment strategies aimed at correcting these dysregulated pathways (45).

In summary, the diverse array of tRNA modifications plays a fundamental role in ensuring the structural integrity and functional efficiency of tRNAs during protein synthesis. These modifications are not only vital for the accurate decoding of mRNA but also serve as regulatory mechanisms that enable cells to respond to environmental changes and stressors. The emerging link between tRNA modification dysregulation and disease underscores the need for further research into the biological significance of these modifications and their potential as therapeutic targets in cancer and other diseases.

### 2.3 Nuclear export and intracellular trafficking of tRNA

**Figure 2:**
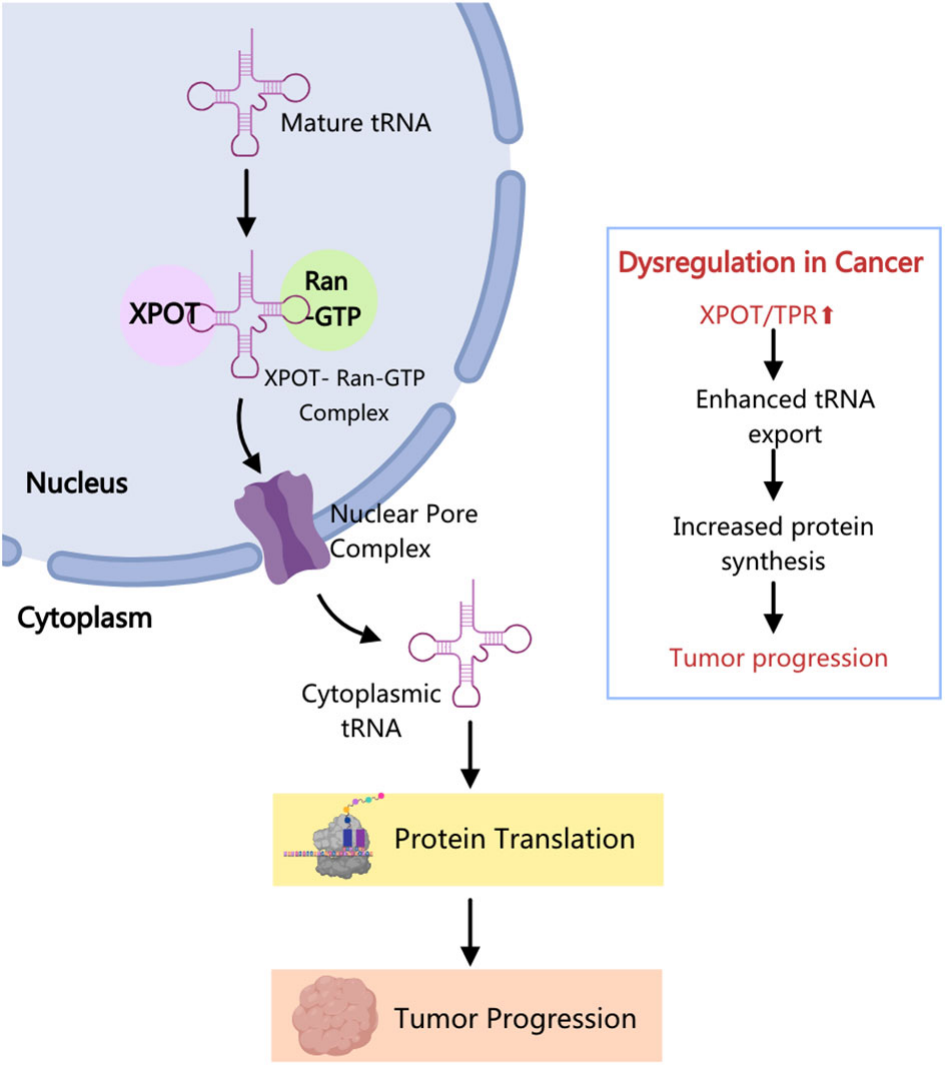
Nuclear export pathway of tRNAs.

The nuclear export of tRNA is a critical process that facilitates the translation of genetic information into proteins, a function essential for cellular homeostasis and proliferation (46, 47). One of the key proteins involved in this process is the nucleoporin TPR (Translocated Promoter Region), which has been identified as a significant regulator of tRNA export in human lung cancer. Studies have demonstrated that TPR is highly expressed in lung cancer tissues, correlating with poor patient prognosis (48). This elevated expression of TPR enhances the nuclear export of tRNA, thereby promoting protein synthesis and supporting the rapid proliferation of cancer cells. Specifically, knockdown experiments of TPR in lung cancer cell lines have shown a marked decrease in tRNA nuclear export, leading to reduced protein synthesis and inhibited cell growth, suggesting that TPR serves as a crucial facilitator of tRNA export in the context of cancer (48). The mechanism by which TPR influences tRNA export appears to involve its interaction with other nuclear export factors, highlighting a complex regulatory network that is pivotal for maintaining the high levels of protein synthesis required in cancerous cells (**Figure 2**).

Mature tRNAs are exported from the nucleus to the cytoplasm through an XPOT- and Ran-GTP-dependent pathway, enabling protein translation. Dysregulated tRNA export may contribute to enhanced protein synthesis and tumor progression.

In addition to TPR, recent findings have implicated the mRNA nuclear export factor NXF1 (Nuclear Export Factor 1) in the nuclear export of tRNA. NXF1, traditionally recognized for its role in mRNA transport, has been shown to associate with tRNAs and facilitate their transport through nuclear pore complexes. This dual functionality of NXF1 underscores a potential overlap between mRNA and tRNA export pathways, suggesting that certain export factors may have broader roles than previously understood (49). The interaction between NXF1 and tRNAs may be particularly important in the context of cellular stress or increased demand for protein synthesis, as it allows for a more efficient and coordinated response to the metabolic needs of the cell. Furthermore, the identification of these regulatory factors not only enhances our understanding of tRNA biogenesis and export but also opens up new avenues for therapeutic interventions targeting these pathways in cancer treatment. The interplay between TPR and NXF1, along with their respective roles in tRNA export, illustrates the intricate regulatory mechanisms that govern tRNA trafficking and highlights the potential for targeting these pathways to disrupt the protein synthesis machinery in cancer cells, thereby offering a novel approach to cancer therapy (49).

## 3 Dysregulation of tRNA networks in cancer

### 3.1 Aberrant tRNA expression across tumor types

The expression profile of tRNA in cancer cells significantly differs from that in normal cells, a phenomenon that has been linked to the altered protein synthesis machinery characteristic of malignancies. In cancerous tissues, specific tRNA species are either upregulated or downregulated, which can profoundly impact the translational landscape and, consequently, the cellular phenotype. For instance, studies have shown that in renal cell cancer (RCC), there is a notable increase in the expression of certain tRNAs, such as tRNA-Glu, tRNA-Gly, and tRNA-Val, while others like tRNA-Gln, tRNA-Leu, and tRNA-Lys are downregulated (50). This shift in tRNA expression can lead to a reprogramming of protein synthesis, favoring the production of proteins that promote cancer cell proliferation and survival. Moreover, the dysregulation of tRNA expression is not merely a byproduct of cancer but appears to play a critical role in tumorigenesis. For example, the upregulation of specific tRNA-derived fragments (tRFs) has been implicated in various cancers, including breast and colorectal cancers, where they contribute to oncogenic signaling pathways and resistance to apoptosis (51, 52).

The overexpression of certain tRNAs in cancer cells is often associated with enhanced cell proliferation and metastatic potential. In prostate cancer, for instance, the tRNA-derived fragment tRF-315 has been shown to protect cancer cells from cisplatin-induced apoptosis, thereby promoting cell survival and tumor progression (53). This fragment’s role in modulating the apoptotic response highlights how specific tRNA species can influence the sensitivity of cancer cells to chemotherapy, thereby impacting treatment outcomes. Additionally, in high-grade serous ovarian cancer, differentially expressed tRFs have been linked to critical pathways involved in cancer progression, suggesting that these tRNA derivatives could serve as potential biomarkers for diagnosis and therapeutic targets (54).

The functional implications of altered tRNA expression extend beyond mere changes in protein synthesis; they also encompass broader regulatory roles in cellular signaling and metabolism. For instance, the expression of tRNA-modifying enzymes has been shown to correlate with cancer aggressiveness, as seen in non-small cell lung cancer (NSCLC), where specific tRNA modifications were significantly downregulated compared to normal tissues (55). This suggests that not only the quantity but also the quality of tRNA modifications plays a crucial role in cancer biology, influencing both the translational efficiency and the fidelity of protein synthesis.

Importantly, changes in total tRNA abundance may not fully reflect the functional reprogramming of protein synthesis in cancer cells. In addition to expression levels, factors such as tRNA charging (aminoacylation) status, codon occupancy, and ribosome dynamics can substantially influence translational output. Recent studies integrating tRNA profiling with ribosome footprinting and aminoacylation analyses have demonstrated that translational efficiency is not always proportional to total tRNA abundance. Therefore, interpretation of cancer-associated tRNA reprogramming should consider both quantitative and functional characteristics of the tRNA pool.

In conclusion, the differential expression of tRNAs between normal and cancer cells is a critical factor in the altered proteomic landscape of tumors. The upregulation of specific tRNAs and their derivatives facilitates oncogenic processes, including enhanced proliferation (56–58), survival (57, 59), and metastasis (60–62). Understanding these differences provides valuable insights into the molecular mechanisms underlying cancer and presents opportunities for the development of novel diagnostic and therapeutic strategies targeting tRNA pathways.

### 3.2 Reprogramming of tRNA pools and codon bias

Cancer cells frequently reshape tRNA expression to match the codon demand of growth-promoting transcripts (63, 64). Because synonymous codons are not used equally, changes in the abundance of specific tRNAs can influence which mRNAs are translated most efficiently (65, 66). This codon usage bias provides a mechanism by which tumors selectively enhance synthesis of proteins required for proliferation, invasion, and metabolic adaptation (67).

Several oncogenes and cell-cycle regulators are enriched for preferred codons that correspond to highly expressed tRNAs in malignant cells (68). By expanding selected tRNA pools, cancer cells can accelerate translation of these transcripts without increasing global protein synthesis to the same extent (34). In contrast, depletion of relevant tRNAs may impair expression of genes involved in differentiation or stress control (69).

This coordinated relationship between codon composition and tRNA availability represents an additional layer of gene regulation in cancer (70). It also suggests that tumor dependence on specific tRNA species may create selective vulnerabilities for therapeutic intervention.

### 3.3 Dysregulated ARSs and tRNA homeostasis

ARSs have emerged as critical players in the landscape of cancer biology, with their upregulation frequently correlating with poor patient prognosis across various cancer types. The role of ARSs extends beyond their traditional function in protein synthesis; they are increasingly recognized for their involvement in oncogenic processes (71). For instance, in gastric cancer, the upregulation of threonyl-tRNA synthetase (TARS) and phenylalanyl-tRNA synthetase (FARSB) has been linked to tumor grade and patient survival outcomes, indicating that ARSs can serve as biomarkers for disease progression and therapeutic targets (72). Similarly, studies have shown that specific ARSs, such as aspartyl-tRNA synthetase (DARS2) and tyrosyl-tRNA synthetase (YARS1), are associated with worse prognoses in hepatocellular carcinoma, highlighting their potential role in tumor initiation and progression (73). Furthermore, the upregulation of ARSs has been documented in various cancers, including lung cancer, where their expression levels are often correlated with increased tumor aggressiveness and metabolic reprogramming, underscoring their importance in supporting the high protein synthesis demands of rapidly proliferating cancer cells (74). Collectively, these findings suggest that ARSs not only facilitate the fundamental processes of protein synthesis but also contribute to the malignant phenotype of tumors, making them critical targets for therapeutic intervention.

In addition to the upregulation of ARSs, the expression of tRNAs exhibits significant heterogeneity in cancer, often showing a decoupling phenomenon from ARS expression. This decoupling indicates that while ARSs may be uniformly elevated in certain cancers, tRNA levels can vary independently, complicating the understanding of their collective roles in tumor biology. For instance, a systematic examination of tRNA and ARS expression across multiple cancer types revealed that while ARSs were predominantly upregulated, the expression of tRNAs was more variable, with some being downregulated in tumors (75). This suggests a complex regulatory mechanism where ARS levels may not always correlate with tRNA availability or function, suggesting that ARS upregulation may support translational buffering under stress and may also reflect translation-independent, noncanonical activities of ARSs in cancer cells. The differential expression of tRNAs may reflect the specific codon usage patterns required for the synthesis of proteins that promote tumor growth and survival, further emphasizing the need to explore the interplay between ARSs and tRNAs in the context of cancer. Moreover, the uncoupling of ARS and tRNA expression raises questions about the regulatory networks governing these molecules and their contributions to tumorigenesis, highlighting an area ripe for further investigation (76). Understanding these dynamics could unveil novel therapeutic strategies that target the specific ARS-tRNA interactions essential for cancer cell proliferation and survival, thereby providing new avenues for cancer treatment.

## 4 Mechanistic roles in tumor progression

### 4.1 Oncogenic tRNA modifications (METTL1/WDR4, m7G)

The role of tRNA methylation in cancer has garnered significant attention in recent years, particularly due to its implications in the regulation of gene translation and tumor progression. One of the key players in this process is the METTL1/WDR4 complex, which mediates the m7G modification of tRNA. This modification has been shown to be crucial for lung cancer progression. In lung cancer cells, elevated levels of m7G-modified tRNAs enhance the translation of oncogenic mRNAs, which are often characterized by a higher frequency of m7G-decoded codons. The mechanistic insights reveal that depletion of METTL1 leads to reduced m7G modification, resulting in decreased cell proliferation, invasion, and overall tumorigenic capabilities both *in vitro* and *in vivo*. Furthermore, the loss of m7G methylation activates stress response pathways, which sensitizes cancer cells to cytotoxic stress, thereby suggesting that the m7G modification not only supports oncogenic translation but also plays a protective role against cellular stressors (55). This underscores the dual role of tRNA methylation in both promoting tumor growth and influencing the stress response, making it a potential therapeutic target.

Additionally, the aberrant activity of tRNA methyltransferases, such as METTL1, has been implicated in translation dysregulation, which is a hallmark of cancer. The dysregulation of tRNA methylation can lead to altered tRNA stability and functionality, thereby affecting the overall protein synthesis landscape within cancer cells. For instance, the upregulation of tRNA methyltransferases has been associated with increased tRNA modifications, which in turn enhances the translation of proteins that drive tumor proliferation, metastasis, and resistance to chemotherapy. This dysregulation is particularly evident in various cancer types, including lung cancer and breast cancer, where the expression levels of tRNA methyltransferases correlate with poor patient prognosis (77, 78). Moreover, the interplay between tRNA modifications and codon usage bias further complicates the translation landscape, as cancer cells may preferentially utilize tRNAs with specific modifications to optimize the translation of growth-promoting mRNAs. This highlights the importance of understanding tRNA methylation not only as a regulatory mechanism of translation but also as a potential biomarker for cancer progression and a target for therapeutic intervention.

In conclusion, the relationship between tRNA methylation and cancer is multifaceted, involving both the promotion of oncogenic translation and the modulation of stress responses. The METTL1/WDR4-mediated m7G modification exemplifies how tRNA modifications can influence tumor biology, presenting opportunities for novel therapeutic strategies aimed at targeting tRNA methylation processes in cancer treatment. Continued research into the specific mechanisms and pathways influenced by tRNA methylation will be critical for developing effective interventions that can disrupt the oncogenic processes driven by dysregulated translation.

### 4.2 TYW2 and translational fidelity

The TYW2 enzyme plays a critical role in the modification of tRNA, particularly in the context of colorectal cancer (CRC). Recent studies have highlighted the epigenetic silencing of TYW2 and its consequential impact on ribosomal frameshifting in CRC. TYW2 is responsible for the synthesis of wybutosine, a modification that stabilizes tRNA and enhances its decoding ability. In CRC, downregulation of TYW2 leads to an accumulation of alternative tRNA modifications, such as imG-14, which has been associated with taxol resistance in cancer cells. Specifically, the substitution of hydroxywybutosine (OHyW) with imG-14 due to TYW2 downregulation has been shown to correlate with the development of taxol resistance in various cancer cell lines (79, 80). This alteration in tRNA modification not only affects the stability and functionality of tRNAs but also plays a pivotal role in the translation process, leading to aberrant protein synthesis that promotes tumor growth and survival. Moreover, the timing of TYW2 downregulation aligns with the onset of taxol resistance, suggesting a potential mechanism by which cancer cells adapt to chemotherapeutic pressures. Therefore, targeting TYW2 or the pathways regulating its expression may provide novel therapeutic strategies to overcome drug resistance in CRC and potentially other malignancies (79, 81).

The Elongator complex, along with CTU and the ALKBH8 family of enzymes, has also emerged as significant players in the landscape of tRNA modifications and their implications in tumor biology. The Elongator complex is essential for the modification of tRNAs, particularly in the context of elongation during protein synthesis. Dysregulation of this complex has been linked to various cancers, where it appears to influence not only the efficiency of translation but also the stability of tRNA molecules. For instance, alterations in the expression of Elongator components have been associated with increased cell proliferation and migration in cancerous tissues. Similarly, CTU, which modifies tRNA at the wobble position, has been implicated in the regulation of tRNA stability and function, thereby impacting the overall translational landscape in tumors. The ALKBH8 family, known for its role in the methylation of tRNA, has also been shown to influence the translation of selenoproteins, which are critical for cellular antioxidant defense mechanisms. Dysregulation of these enzymes can lead to an imbalance in tRNA modifications, resulting in impaired protein synthesis and contributing to tumorigenesis. Collectively, these findings underscore the importance of tRNA modification enzymes as potential biomarkers and therapeutic targets in cancer, highlighting their multifaceted roles in regulating translation and cellular homeostasis (82, 83).

### 4.3 ARS–tRNA decoupling and noncanonical functions

**Figure 3:**
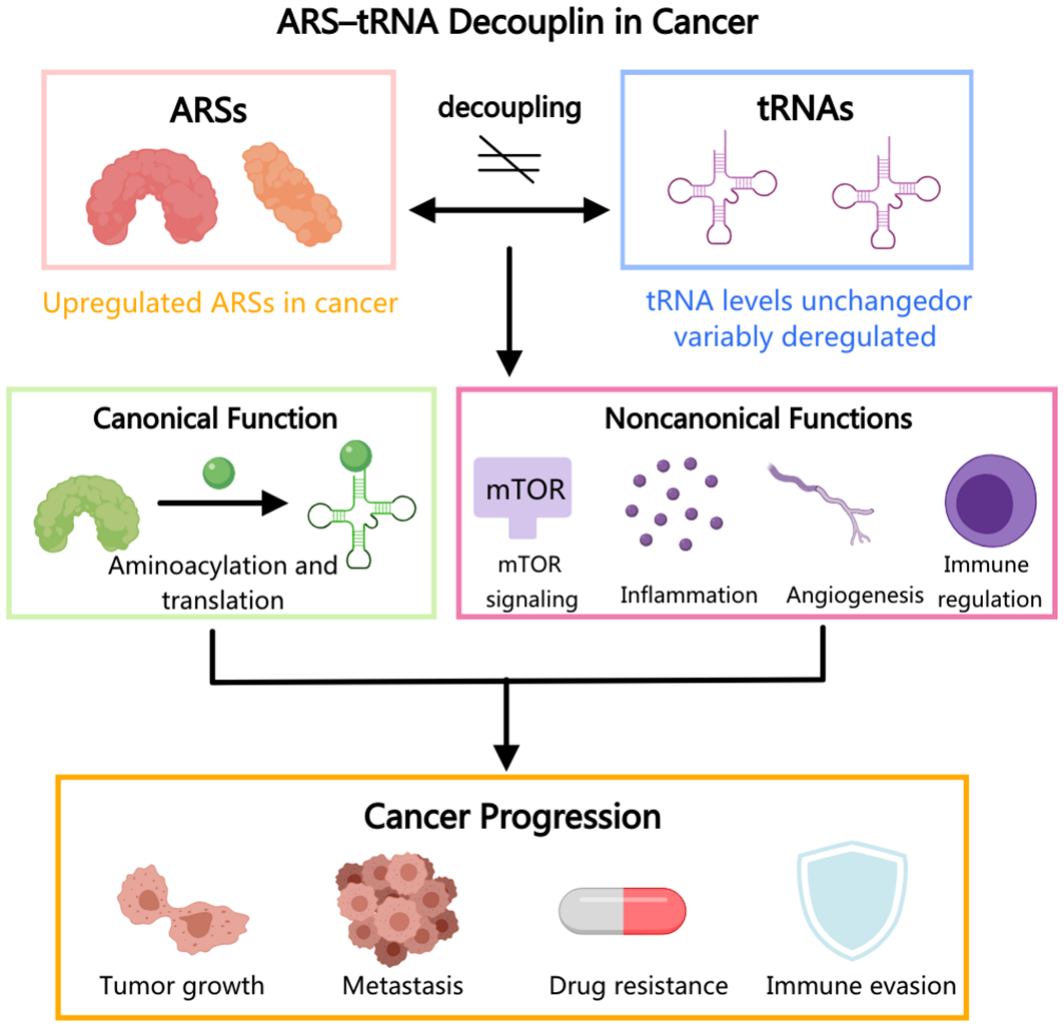
ARS–tRNA decoupling and noncanonical functions in cancer.

Although aminoacyl-tRNA synthetases (ARSs) and tRNAs act together during protein translation, their expression is not always coordinated in cancer. Pan-cancer analyses have shown that many ARSs are upregulated, whereas tRNA levels vary considerably among tumor types. This pattern suggests that increased ARS expression is not solely a consequence of higher translational demand (**Figure 3**).

In cancer cells, ARSs are frequently upregulated independently of tRNA abundance. Beyond aminoacylation, ARSs participate in signaling pathways related to metabolism, inflammation, angiogenesis, and immune regulation.

One possible explanation is the need to maintain translation under cellular stress. During hypoxia, nutrient deprivation, or anticancer treatment, higher ARS expression may help preserve aminoacylation efficiency and protein synthesis, thereby supporting tumor cell survival.

In addition, ARSs possess functions beyond aminoacylation. Several members of this family participate in pathways related to mTOR signaling, inflammation, angiogenesis, and immune regulation. Some ARSs or their cleavage products can also modulate the tumor microenvironment in a cytokine-like manner.

Another possibility is selective translational reprogramming. Tumor cells often favor translation of transcripts enriched for specific codons. Upregulation of certain ARSs may facilitate efficient decoding of these transcripts without requiring a parallel increase in total tRNA abundance.

These findings indicate that ARSs may serve as independent biomarkers or therapeutic targets. Further studies combining transcriptomic and functional approaches are needed to define how ARS–tRNA imbalance contributes to tumor progression and treatment resistance.

### 4.4 Stress adaptation and codon-specific translation

The role of tRNA modifications, particularly m7G, in regulating stress responses in tumor cells has garnered significant attention for its implications in cancer biology. The m7G modification is crucial for the stability and function of tRNAs, protecting them from stress-induced cleavage that can lead to the generation of tRNA-derived fragments (tRFs) and tRNA halves (tiRNAs) (84). Under stress conditions, such as heat shock or oxidative stress, the integrity of tRNA molecules is compromised, which can trigger their cleavage and subsequently alter the cellular stress response pathways. The presence of m7G on tRNAs enhances their resilience against such stressors, thereby supporting the survival of tumor cells under adverse conditions. For instance, studies have shown that m7G modification promotes the translation of specific oncogenes, such as SLUG and SNAIL, under heat stress, facilitating tumor metastasis and progression (84). This modification acts as a regulatory mechanism that not only stabilizes tRNA but also influences the translational efficiency of mRNA transcripts that are critical for cell survival during stress. Conversely, the absence of m7G modification can lead to increased susceptibility of tRNAs to cleavage, resulting in the production of tiRNAs and tRFs that can activate stress pathways, potentially leading to apoptosis or senescence in tumor cells (85). This dual role of tRNA modifications highlights the delicate balance that tumor cells must maintain to adapt to fluctuating environmental stresses while promoting their own survival and proliferation. The dysregulation of tRNA modifications, such as m7G, can thus serve as a biomarker for cancer progression and a potential therapeutic target, as manipulating these modifications may enhance the effectiveness of existing treatments or lead to the development of novel therapeutic strategies aimed at reprogramming the stress response in cancer cells (13, 15, 86–90).

Moreover, the generation of tRNA fragments, particularly under conditions of modification deficiency, underscores the importance of these modifications in maintaining cellular homeostasis. For example, the demethylation of tRNA by enzymes such as ALKBH1 has been shown to destabilize tRNA structures, leading to increased cleavage and the production of tRFs that can modulate gene expression through mechanisms similar to those of microRNAs (85). These tRFs can engage in post-transcriptional regulation, influencing the expression of genes involved in cell cycle control, apoptosis, and stress responses, thereby contributing to the complex interplay between tRNA modifications and tumor cell fate (91). The ability of tRNA-derived fragments to regulate gene expression and cellular responses during stress highlights their potential as therapeutic targets in cancer treatment. Understanding the intricate mechanisms by which tRNA modifications and their resultant fragments influence tumor biology will be critical in developing strategies to exploit these pathways for therapeutic gain. Overall, the regulation of tRNA modifications presents a promising avenue for enhancing our understanding of cancer biology and improving clinical outcomes through targeted therapies that manipulate these critical molecular players in tumor cell stress responses.

## 5 tsRNAs in cancer biology

### 5.1 Classification and biogenesis

**Figure 4:**
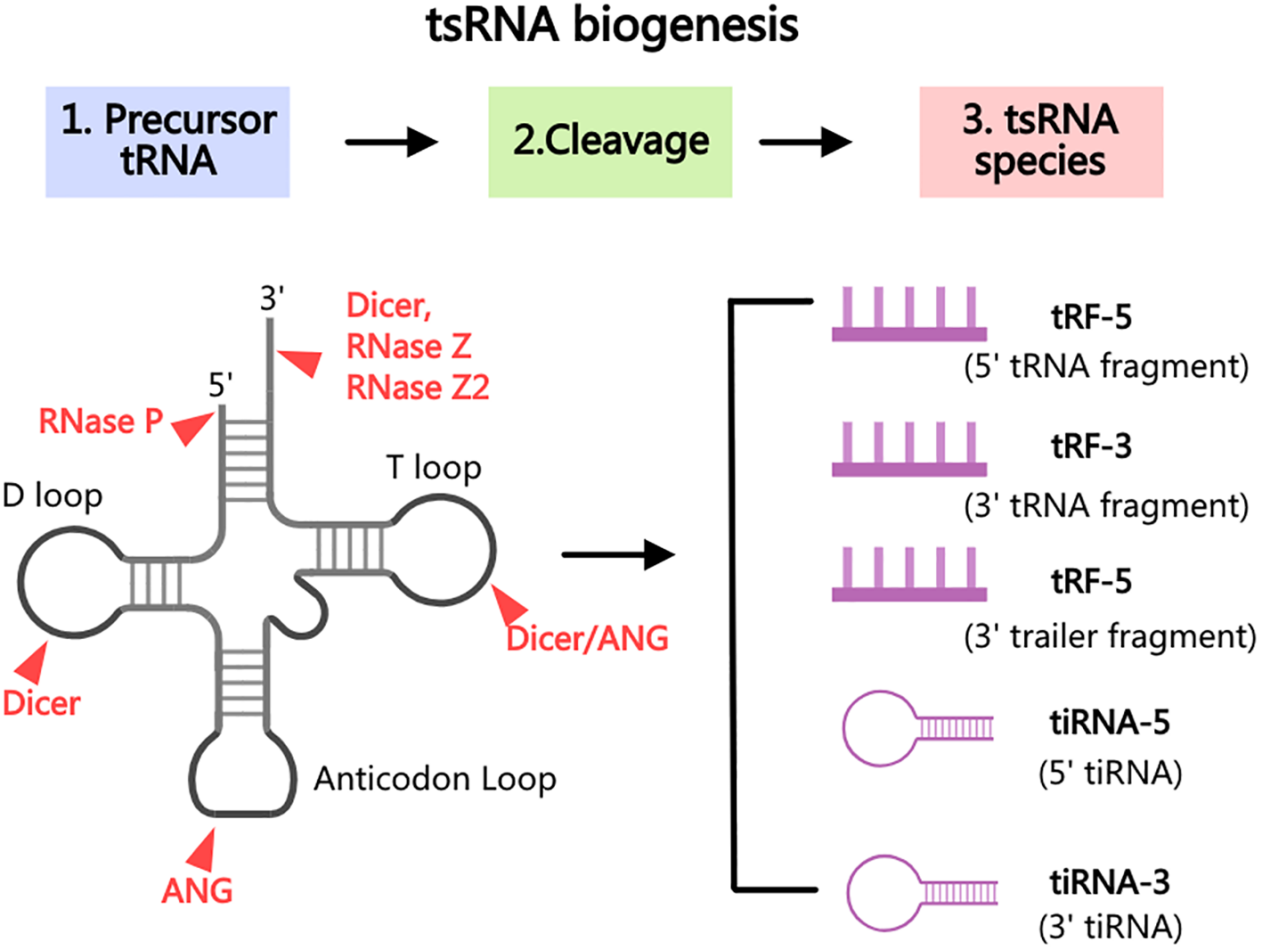
Biogenesis and classification of tsRNAs.

Transfer RNA-derived small RNAs (tsRNAs) are a newly recognized class of non-coding RNAs that have garnered significant attention due to their diverse roles in cellular regulation and disease processes (92, 93). TsRNAs can be classified into two primary categories based on their biogenesis: tRNA halves (tiRNAs) and tRNA-derived fragments (tRFs). TiRNAs are typically generated through the cleavage of mature tRNAs at the anticodon loop, resulting in two equal halves of the tRNA molecule (94). This cleavage is primarily facilitated by specific ribonucleases, notably angiogenin and Dicer, which act under stress conditions or in response to various cellular stimuli (95). On the other hand, tRFs are produced through different cleavage sites on tRNAs, leading to fragments of varying lengths, which can be classified further based on their specific origin within the tRNA structure. For instance, tRFs can be categorized into tRF-1s, tRF-2s, tRF-3s, tRF-5s, and i-tRFs, each defined by the specific nucleotide positions from which they are derived (96). This classification reflects the complex nature of tsRNA biogenesis, which is influenced by various factors, including the type of stress experienced by the cell and the specific ribonucleases involved in the cleavage process (**Figure 4**).

Mature and precursor tRNAs can be cleaved by specific ribonucleases to generate tiRNAs and multiple classes of tRNA-derived fragments (tRFs), including tRF-1, tRF-3, tRF-5, and i-tRFs.

The biogenesis of tsRNAs is not merely a result of random degradation of tRNA molecules; rather, it is a tightly regulated process that is influenced by the cellular environment and specific stimuli. For example, under stress conditions, such as oxidative stress or nutrient deprivation, the expression of certain ribonucleases like angiogenin increases, leading to the enhanced production of tiRNAs and tRFs (97). This suggests that tsRNAs play a crucial role in cellular stress responses, acting as regulatory molecules that can modulate gene expression and cellular function. Moreover, the production of tsRNAs is also subject to post-transcriptional modifications, which can affect their stability and functionality, further complicating their biogenesis (98). The precise cleavage of tRNAs by these enzymes indicates a sophisticated regulatory mechanism that allows cells to adapt to changing conditions, highlighting the potential of tsRNAs as biomarkers for various diseases, including cancer and metabolic disorders (99).

In summary, tsRNAs represent a fascinating area of research within the field of molecular biology, with their classification into tiRNAs and tRFs underscoring the complexity of their biogenesis. The involvement of specific ribonucleases in their production and the influence of cellular stressors on their expression patterns suggest that tsRNAs are not merely byproducts of tRNA degradation but rather critical players in the regulation of gene expression and cellular homeostasis. As our understanding of tsRNA biology expands, it is likely that these molecules will emerge as important targets for therapeutic intervention and diagnostic applications in various diseases.

### 5.2 Regulation of proliferation and metastasis

Transfer RNA-derived small RNAs (tsRNAs) have emerged as crucial regulators in the context of cancer, particularly in tumor cell proliferation and metastasis (100). These small non-coding RNAs are generated through the cleavage of precursor tRNAs and play significant roles in various biological processes, including gene expression regulation, protein translation, and cellular signaling pathways. One of the primary mechanisms by which tsRNAs exert their influence is through the modulation of protein synthesis. For instance, tsRNAs can interact with ribosomal machinery and affect the translation of specific mRNAs, leading to altered protein levels that drive tumor growth and metastasis. Additionally, tsRNAs have been shown to regulate key signaling pathways associated with cancer progression, such as the PI3K/AKT and Wnt/β-catenin pathways, both of which are critical for cell survival, proliferation, and migration (101, 102). The dysregulation of these pathways due to abnormal tsRNA expression can disrupt normal cellular functions and promote oncogenic processes, including enhanced cell proliferation and the ability of cancer cells to invade surrounding tissues. Furthermore, tsRNAs can also influence the expression of genes involved in epithelial-mesenchymal transition (EMT), a critical process that facilitates metastasis by allowing cancer cells to acquire migratory and invasive properties (56, 103).

The expression profiles of tsRNAs vary significantly across different cancer types, indicating a complex functional landscape that may reflect the unique biological contexts of various malignancies. For example, specific tsRNAs have been identified as either upregulated or downregulated in different cancers, correlating with distinct clinicopathological features such as tumor stage, lymph node metastasis, and patient prognosis (35, 104). In gastric cancer, for instance, certain tsRNAs have been implicated in promoting cell proliferation and invasion, while others appear to function as tumor suppressors, highlighting their dual roles depending on the tumor microenvironment and genetic background. This functional divergence underscores the potential of tsRNAs as biomarkers for cancer diagnosis and prognosis, as well as their promise as therapeutic targets.

Moreover, the clinical relevance of tsRNAs is further supported by their stability and abundance in body fluids, making them attractive candidates for non-invasive liquid biopsy approaches (105, 106). Research has demonstrated that specific tsRNAs can serve as biomarkers for early detection of tumors, monitoring treatment responses, and predicting outcomes in patients. For example, elevated levels of certain tsRNAs have been associated with poor prognosis in lung cancer and gastric cancer, suggesting that they may reflect the underlying tumor biology and metastatic potential (107).

In conclusion, tsRNAs play a multifaceted role in the regulation of tumor cell proliferation and metastasis through their influence on protein translation, gene expression, and signaling pathways. The variability in tsRNA expression across different cancers not only highlights their potential as biomarkers but also emphasizes the need for further research to elucidate their precise mechanisms of action and to explore their therapeutic applications in oncology. As our understanding of tsRNAs continues to evolve, they may well become integral components of cancer diagnostics and targeted therapies, paving the way for more personalized treatment strategies in cancer care.

### 5.3 Immune modulation and signaling crosstalk

Accumulating evidence indicates that tsRNAs are involved in tumor immunity and intercellular communication, in addition to their roles in proliferation and metastasis (19, 108, 109). Similar to microRNAs, a subset of tsRNAs can associate with Argonaute proteins and enter RNA-induced silencing complexes, thereby regulating transcripts related to inflammatory signaling, immune responses, and tumor progression (110, 111). These findings support a sequence-dependent post-transcriptional regulatory role for selected tsRNAs.

Extracellular tsRNAs also function within the tumor microenvironment. Tumor-derived exosomal tsRNAs mediate communication between cancer cells and surrounding stromal or immune cells, and have been linked to fibroblast activation, macrophage polarization, and local immunosuppression (112–115). Such effects may promote metastatic niche formation and tumor adaptation.

In parallel, tsRNAs have been implicated in innate immune activation through Toll-like receptors (TLRs), particularly endosomal RNA-sensing receptors (116). Depending on the cellular context, this signaling can trigger inflammatory cytokine production or immunosuppressive programs, indicating that tsRNA-mediated immune responses are context dependent.

tsRNAs may also contribute to immune evasion. Reported mechanisms include modulation of PD-L1-related pathways and suppression of antitumor immune-cell activity (117–119). Although this field is still developing, tsRNAs are increasingly recognized as potential regulators of tumor–immune interactions and may influence responses to immunotherapy.

### 5.4 Biomarker potential

tsRNAs have emerged as significant players in cancer biology, particularly in their roles as potential diagnostic and prognostic biomarkers across various malignancies, including colorectal cancer and lung cancer. Recent studies have highlighted the association between the expression levels of specific tsRNAs and the metastatic potential and malignancy of tumors. For instance, in colorectal cancer, certain tsRNAs have been identified with differential expression profiles that correlate with tumor stage, lymph node involvement, and overall patient prognosis. A study demonstrated that a panel of four differentially expressed tsRNAs could effectively distinguish between cancerous and normal tissues, achieving a diagnostic accuracy with an area under the curve (AUC) of 0.97 in validation datasets (120). Similarly, in lung cancer, elevated levels of specific tsRNAs in serum samples were linked to poor prognostic outcomes, with a meta-analysis indicating a high diagnostic odds ratio (DOR) of 16.56, suggesting their robust potential as biomarkers for early detection and prognosis (121). This correlation between tsRNA expression and cancer severity underscores their utility in clinical settings, where they could aid in stratifying patients based on risk and guiding therapeutic decisions.

Moreover, the stability of tsRNAs in bodily fluids such as blood and serum enhances their clinical applicability as biomarkers. Unlike many other RNA species, tsRNAs exhibit remarkable stability, which is crucial for their detection in non-invasive liquid biopsies. This characteristic allows for the development of sensitive assays that can accurately measure tsRNA levels in patients, providing a window into the tumor’s biological behavior without the need for invasive tissue biopsies. For instance, a novel study utilized high-throughput sequencing to identify specific tsRNAs that were significantly upregulated in the serum of lung adenocarcinoma patients, demonstrating their potential as non-invasive biomarkers for diagnosis and monitoring disease progression (122). Additionally, the use of advanced techniques such as reverse transcription quantitative polymerase chain reaction (RT-qPCR) has validated the presence of these tsRNAs in clinical samples, further supporting their role in cancer diagnostics (104). The ability to detect these molecules in serum not only facilitates early diagnosis but also enables ongoing monitoring of therapeutic responses and disease recurrence.

In conclusion, the emerging evidence supports the potential of tsRNAs as reliable diagnostic and prognostic biomarkers in cancer. Their association with tumor progression, coupled with their stability in bodily fluids, positions them as valuable tools in the clinical management of cancers such as colorectal and lung cancer. Future research should focus on standardizing the methodologies for tsRNA detection and validation in larger cohorts to fully establish their clinical utility and integrate them into routine diagnostic workflows. This could ultimately lead to improved patient outcomes through personalized treatment strategies based on tsRNA profiling.

To provide a concise comparison of representative tRNA-related alterations discussed in this review, we summarized their cancer associations, key mechanisms, evidence levels, and reported biomarker performance in **Table 1**.

**Table 1:** Representative tRNA-related alterations in cancer: mechanisms, evidence, and biomarker potential.

Component	Cancer association	Key mechanism	Evidence level	Biomarker AUC
METTL1 (m7G)	Lung cancer/HCC	Oncogenic translation	Medium (xenograft)	–
NSUN2 (m5C)	Breast/CRC	tRNA stabilization	Medium	–
TYW2	CRC	Translational fidelity/drug resistance	Medium	–
tsRNA panel	CRC	Proliferation/EMT	High (clinical cohort)	0.97
Serum tsRNAs	Lung cancer	Circulating biomarker	High	Variable
TPR export	Lung cancer	Protein synthesis	Low (correlation)	–
XPOT	TNBC	Selective tRNA export	Medium	–
ARSs	Multiple cancers	Stress adaptation/signaling	Medium	–

## 6 Therapeutic opportunities

### 6.1 Targeting tRNA modification enzymes

tRNA-modifying enzymes have emerged as promising therapeutic targets in cancer because they regulate translational efficiency, stress adaptation, and codon-biased protein synthesis (21, 34, 123). Compared with global translation inhibitors, targeting specific tRNA modification pathways may more selectively disrupt tumor cells that depend on adaptive translational programs.

The METTL1/WDR4 complex catalyzes N7-methylguanosine (m7G) modification in tRNAs and is frequently upregulated in multiple cancers. METTL1-driven m7G modification promotes translation of oncogenic transcripts involved in proliferation, metastasis, and metabolic reprogramming (124–128). Inhibition of METTL1 has shown anti-tumor effects in hepatocellular carcinoma, lung cancer, and prostate cancer, highlighting its therapeutic potential.

ALKBH1 functions as a tRNA demethylase and participates in stress-responsive translational regulation (56, 129). By remodeling tRNA methylation states, ALKBH1 can enhance tumor cell survival under oxidative stress or drug exposure. Recent studies suggest that ALKBH1 contributes to tumor progression and therapy resistance, making it a potential sensitization target.

NSUN2 catalyzes 5-methylcytosine (m5C) modification in tRNAs and is associated with increased RNA stability and aggressive tumor behavior (130–133). NSUN2-mediated methylation also protects tRNAs from stress-induced cleavage, thereby sustaining protein synthesis in hostile tumor microenvironments.

Despite these advances, therapeutic challenges remain, including potential toxicity to normal tissues and compensatory RNA-modification pathways. Future strategies will likely depend on biomarker-guided selection and combination therapies. Overall, tRNA modification enzymes represent a promising class of translational targets for precision oncology.

### 6.2 Targeting tsRNAs

The role of tsRNAs in cancer has garnered significant attention as they emerge as potential therapeutic targets. Recent studies have demonstrated that specific tsRNAs can modulate tumor cell growth and drug resistance, highlighting their importance in cancer biology. For instance, tsRNAs have been shown to influence the proliferation and migration of various cancer cell types, including breast, gastric, and NSCLC cells. One notable example is the tRF-20-M0NK5Y93, which has been identified as significantly downregulated in NSCLC tissues. Its overexpression was found to inhibit cell proliferation and migration while promoting apoptosis, indicating its potential as a tumor suppressor (134). Furthermore, tsRNAs can interact with key signaling pathways, such as the Wnt/β-catenin pathway, to exert their effects on tumor growth and metastasis. For instance, the tiRNA-Val-CAC-001 has been shown to target LRP6, a crucial component of the Wnt pathway, thereby inhibiting gastric cancer cell proliferation and metastasis (134). These findings underscore the therapeutic potential of targeting tsRNAs to modulate cancer progression and enhance treatment efficacy.

In addition to their role in tumor growth, tsRNAs are also implicated in mediating RNA interference and immune regulation, further expanding their therapeutic potential (19, 135, 136). tsRNAs can interact with Argonaute proteins, similar to microRNAs, to regulate gene expression through RNA silencing mechanisms (137–139). This interaction allows tsRNAs to influence various biological processes, including cell cycle regulation, apoptosis, and immune responses (95). For example, specific tsRNAs have been shown to modulate the immune microenvironment, potentially enhancing the efficacy of immunotherapies (115, 140, 141). Moreover, the stability of tsRNAs in bodily fluids makes them attractive candidates for liquid biopsy applications, providing a non-invasive means to monitor treatment responses and disease progression (142).

The exploration of tsRNAs as therapeutic targets is still in its early stages, and several challenges remain. Understanding the precise mechanisms by which tsRNAs exert their effects in different cancer types is crucial for developing targeted therapies. Additionally, the development of delivery systems for tsRNA-based therapeutics, such as nanoparticles or exosome-based platforms, is essential to enhance their bioavailability and efficacy in clinical settings (143). As research continues to unravel the complex roles of tsRNAs in cancer, their potential as diagnostic biomarkers and therapeutic agents will likely expand, paving the way for innovative treatment strategies that harness the unique properties of these small RNAs. The identification of specific tsRNAs that correlate with tumor characteristics and treatment outcomes will be critical for the successful integration of tsRNA-based therapies into clinical practice, ultimately improving patient management and outcomes in cancer care.

### 6.3 Disrupting nuclear export pathways

The process of tRNA nuclear export is crucial for maintaining the balance of protein synthesis within cells, particularly in cancerous tissues where the demand for protein production is significantly heightened. Abnormalities in tRNA nuclear export can lead to a reduction in the availability of aminoacylated tRNAs in the cytoplasm, which in turn impairs protein synthesis and inhibits tumor cell proliferation. Research has shown that the nuclear export of tRNAs is a tightly regulated mechanism, and disruptions in this process can have profound effects on cellular functions. For instance, the nucleoporin TPR has been identified as a key player in promoting tRNA nuclear export; its expression is notably increased in lung cancer tissues and correlates with poor patient prognosis (48). When TPR is knocked down in lung cancer cell lines, there is a marked decrease in tRNA export, leading to diminished protein synthesis and reduced cell growth. This suggests that the efficient export of tRNAs is not merely a cellular housekeeping function but is integral to the aggressive proliferation of cancer cells. Similarly, in triple-negative breast cancer (TNBC), the nuclear export protein receptor XPOT has been shown to orchestrate the export of specific tRNAs, which are critical for the proliferation of TNBC cells. Knockdown of XPOT results in inhibited cell proliferation and altered cytokinesis, underscoring the importance of tRNA export in cancer cell growth (144). Thus, the disruption of tRNA nuclear export can impede the protein synthesis machinery of cancer cells, providing a potential avenue for therapeutic intervention.

The regulation of tRNA nuclear export represents a potential new target for cancer therapy. Given the critical role that tRNA export plays in sustaining the high levels of protein synthesis required for tumor growth, strategies aimed at modulating this process could yield novel therapeutic approaches. For instance, targeting the proteins involved in tRNA nuclear export, such as TPR and XPOT, could inhibit cancer cell proliferation by disrupting their protein synthesis capabilities. This is particularly relevant in the context of cancers that exhibit heightened dependency on specific tRNAs for their growth and survival. The potential for pharmacological agents that can selectively inhibit these export pathways offers a unique strategy to starve tumors of the essential proteins they require for rapid growth. Furthermore, the identification of specific tRNA isodecoders that are preferentially exported and utilized by cancer cells opens the door to precision medicine approaches, where therapies could be tailored to target the unique tRNA profiles of individual tumors. As research continues to elucidate the mechanisms governing tRNA nuclear export and its implications in cancer biology, the development of therapeutics that exploit these pathways may become a cornerstone of cancer treatment strategies (145, 146). By understanding and manipulating the regulatory networks surrounding tRNA export, it may be possible to create effective interventions that can halt or even reverse cancer progression.

### 6.4 ARS inhibitors

Aminoacyl-tRNA synthetases (ARSs) have emerged as promising targets for cancer therapy due to their multifaceted roles beyond their canonical function in protein synthesis. Recent studies have highlighted the development of ARS inhibitors and their potential anti-tumor activities. For instance, the inhibition of specific ARSs has been shown to suppress tumor growth in various cancer models, suggesting that targeting these enzymes could disrupt the metabolic reprogramming that cancer cells undergo to sustain their rapid proliferation (72, 147). Furthermore, the interplay between ARSs and cellular stress responses has been identified as a critical factor in tumorigenesis. ARSs not only facilitate the aminoacylation of tRNAs but also engage in non-canonical functions that influence cellular signaling pathways, apoptosis, and immune responses. For example, tryptophanyl-tRNA synthetase has been implicated in modulating immune responses by interacting with interferon pathways, thereby affecting cancer cell survival and proliferation (147). This dual role of ARSs in both translation and regulatory functions makes them attractive candidates for therapeutic intervention, as inhibiting their activity could simultaneously target the growth and survival pathways of cancer cells.

Moreover, the role of ARSs in cancer cell stress and metabolic reprogramming is gaining attention. Cancer cells often experience various forms of stress, including nutrient deprivation and oxidative stress, which can trigger adaptive responses mediated by ARSs. For example, studies have shown that ARSs can regulate the integrated stress response (ISR), a protective mechanism that allows cells to survive under adverse conditions (148). This regulation is crucial in the context of cancer, where cells must adapt to fluctuating microenvironments. The upregulation of ARSs in tumor cells has been associated with enhanced protein synthesis and cell survival, contributing to tumor progression and resistance to therapy (149, 150). Thus, targeting ARSs could not only inhibit tumor growth but also sensitize cancer cells to conventional therapies by disrupting their adaptive stress responses.

In addition to their role in stress responses, ARSs are involved in metabolic pathways that are frequently altered in cancer. For instance, the aminoacyl-tRNA biosynthesis pathway has been identified as significantly upregulated in various cancers, including gastric cancer, where specific ARSs correlate with tumor grade and patient prognosis (72). This metabolic reprogramming is essential for supporting the high demands of protein synthesis in rapidly dividing cancer cells. Therefore, the development of ARS inhibitors could provide a novel strategy to target the metabolic vulnerabilities of cancer cells, potentially leading to improved therapeutic outcomes.

The current landscape of ARS inhibitor development is promising, with several compounds showing efficacy in preclinical models. For instance, novel small-molecule inhibitors targeting specific ARSs have demonstrated selective cytotoxicity towards cancer cells while sparing normal cells (151). This selectivity is particularly important in cancer therapy, as it may reduce the side effects commonly associated with conventional chemotherapeutics. Furthermore, ongoing clinical trials are exploring the combination of ARS inhibitors with other therapeutic modalities, such as immune checkpoint inhibitors and targeted therapies, to enhance their efficacy and overcome resistance mechanisms (148).

In conclusion, the potential of ARSs as therapeutic targets in cancer treatment is underscored by their critical roles in protein synthesis, metabolic reprogramming, and stress response regulation. Continued research into ARS inhibitors and their mechanisms of action will be essential for translating these findings into effective clinical strategies for cancer therapy. The multifaceted nature of ARSs offers a unique opportunity to develop innovative treatments that can address the complexities of cancer biology and improve patient outcomes.

## 7 Methodological challenges and future perspectives

### 7.1 Challenges in tRNA profiling

The exploration of the molecular mechanisms underlying the nuclear export pathways of tRNA represents a critical area for future research in cancer biology. Recent studies have highlighted the significance of tRNA modifications and their roles in various cellular processes, including translation regulation and stress responses (152). Understanding how tRNAs are exported from the nucleus to the cytoplasm is essential, as this process is tightly linked to protein synthesis and cellular growth, both of which are dysregulated in cancer. For instance, the nuclear basket proteins Mlp1 and Mlp2 have been identified as key regulators of tRNA export, and their expression levels have been correlated with cancer prognosis (48). Future research should focus on elucidating the detailed molecular interactions and regulatory networks involved in tRNA nuclear export, particularly in the context of tumorigenesis. Investigating the impact of oncogenic signaling pathways on tRNA export mechanisms may reveal novel therapeutic targets that can be exploited to disrupt the aberrant protein synthesis that characterizes many cancers. Additionally, the potential role of tRNA-derived fragments (tRFs) in modulating these pathways should be further explored, as their dysregulation has been implicated in cancer progression (63).

Several specialized sequencing approaches have been developed to profile tRNAs and tsRNAs, including hydro-tRNA-seq, DM-tRNA-seq, ARM-seq, YAMAT-seq, and mim-tRNA-seq. These methods were designed to address technical barriers that limit standard RNA-seq-based tRNA quantification. The compact secondary structure of mature tRNAs can reduce reverse-transcription efficiency and introduce library-construction bias. In addition, abundant post-transcriptional modifications, such as m1A and m3C, can cause reverse-transcription stops or misincorporation events, leading to inaccurate quantification. Further challenges include incomplete removal of modification-induced biases, mapping ambiguity among highly similar tRNA genes and isodecoders, and difficulty distinguishing mature tRNAs from tRNA-derived fragments. Therefore, differential tRNA expression data should be interpreted with caution, particularly when comparing datasets generated using different sequencing or preprocessing strategies.

### 7.2 Precision therapeutic strategies

Furthermore, the development of precision therapeutic strategies based on the regulation of tRNA nuclear export holds great promise for cancer treatment. As the understanding of tRNA modifications and their influence on translation efficiency expands, researchers can begin to design targeted therapies that specifically modulate these pathways. For example, targeting the enzymes responsible for tRNA modifications, such as METTL1, has been shown to influence cancer cell proliferation and survival. By leveraging the unique characteristics of tRNA modifications and their roles in cancer biology, it may be possible to develop precision therapies that enhance the efficacy of existing treatments or overcome resistance mechanisms. Moreover, the identification of biomarkers related to tRNA modifications could facilitate the stratification of patients for personalized treatment approaches, allowing for more effective interventions tailored to individual tumor profiles (153).

### 7.3 Clinical outlook

In summary, future research should prioritize the elucidation of tRNA nuclear export mechanisms and the development of targeted therapeutic strategies that exploit these pathways. The integration of tRNA biology into the broader context of cancer research may yield significant advancements in understanding tumorigenesis and improving clinical outcomes for cancer patients.

## 8 Discussion

The intricate world of tRNA and its myriad of chemical modifications plays a pivotal role in maintaining translational accuracy and cellular homeostasis (1, 2). Recent research has underscored the association of tRNA dysregulation with the onset and progression of various cancers (10, 14, 16, 62, 154–156). As we have explored in this review, the aberrant expression and functional alterations of tRNA modification enzymes, tRNA-derived small RNAs, and aminoacyl-tRNA synthetases have been associated with tumorigenesis, suggesting potential avenues for diagnostic and therapeutic interventions.

From an expert perspective, it is essential to recognize the multifaceted nature of tRNA biology and its implications in cancer. The specific expression patterns of tRNA-related molecules in different cancer types highlight the need for a nuanced understanding of their roles (157–159). For instance, the differential regulation of tRNA modification enzymes can lead to altered protein synthesis profiles that favor cancer cell survival and proliferation. This specificity not only enhances our understanding of cancer biology but also suggests opportunities for the development of targeted therapies that exploit these unique molecular signatures.

Moreover, the regulation of tRNA nuclear export mechanisms offers an additional perspective for understanding cancer-related protein synthesis (63, 160, 161). The involvement of factors such as TPR and NXF1 in the nuclear-cytoplasmic transport of tRNA and their functional abnormalities in tumor progression highlight the regulatory roles of tRNA in cancer (48, 144). This emerging perspective encourages a more integrated approach to studying cancer, where the interplay between tRNA modifications, nuclear export, and protein synthesis is considered holistically.

Looking ahead, future research must delve deeper into the specific molecular mechanisms underlying tRNA modifications and the roles of tRNA-derived fragments in cancer (162, 163). Understanding these processes at a granular level will be crucial for translating these findings into clinical applications. The potential for tRNA modifications and their derivatives to serve as biomarkers for early cancer diagnosis and prognosis is being actively investigated. Furthermore, elucidating their roles in targeted therapies could inform the development of personalized medicine strategies.

In conclusion, the comprehensive exploration of the tRNA regulatory network represents a developing area in cancer research. By synthesizing insights from various research perspectives, we can foster a more cohesive understanding of how tRNA modifications contribute to cancer biology. This integrative approach advances our knowledge of the molecular underpinnings of cancer and may inform the development of innovative therapeutic strategies. As we continue to unravel the complexities of tRNA biology, further advancements in cancer treatment and precision medicine may become possible. The journey ahead is one of collaboration and discovery, where the convergence of basic and clinical research will illuminate new pathways for combating cancer.
